# Understanding intra-neighborhood patterns in PM_2.5_ and PM_10_ using mobile monitoring in Braddock, PA

**DOI:** 10.1186/1476-069X-11-76

**Published:** 2012-10-10

**Authors:** Brett J Tunno, Kyra Naumoff Shields, Paul Lioy, Nanjun Chu, Joseph B Kadane, Bambang Parmanto, Gede Pramana, Jennifer Zora, Cliff Davidson, Fernando Holguin, Jane E Clougherty

**Affiliations:** 1 Department of Environmental and Occupational Health, University of Pittsburgh Graduate School of Public Health, Pittsburgh, Pennsylvania, USA; 2 Department of Pediatric Pulmonology and Pediatric Environmental Medicine Center, University of Pittsburgh Medical Center, Pittsburgh, Pennsylvania, USA; 3 University of Pittsburgh Health Information Management, Pittsburgh, Pennsylvania, USA; 4 Department of Statistics, Carnegie Mellon University, Pittsburgh, Pennsylvania, USA; 5 Emory University, Atlanta, Georgia, USA; 6 Syracuse University, Syracuse, New York, USA; 7 Environmental and Occupational Health Sciences Institute (EOHSI), Robert Wood Johnson Medical School (RWJMS), Piscataway, New York, USA

**Keywords:** “Particulate matter”, “Mobile monitoring”, “Spatial and temporal variability”, “Braddock, Pennsylvania”

## Abstract

**Background:**

Braddock, Pennsylvania is home to the Edgar Thomson Steel Works (ETSW), one of the few remaining active steel mills in the Pittsburgh region. An economically distressed area, Braddock exceeds average annual (>15 μg/m^3^) and daily (>35 μg/m^3^) National Ambient Air Quality Standards (NAAQS) for particulate matter (PM_2.5_).

**Methods:**

A mobile air monitoring study was designed and implemented in morning and afternoon hours in the summer and winter (2010–2011) to explore the within-neighborhood spatial and temporal (within-day and between-day) variability in PM_2.5_ and PM_10_.

**Results:**

Both pollutants displayed spatial variation between stops, and substantial temporal variation within and across study days. For summer morning sampling runs, site-specific mean PM_2.5_ ranged from 30.0 (SD = 3.3) to 55.1 (SD = 13.0) μg/m^3^. Mean PM_10_ ranged from 30.4 (SD = 2.5) to 69.7 (SD = 51.2) μg/m^3^, respectively. During summer months, afternoon concentrations were significantly lower than morning for both PM_2.5_ and PM_10_, potentially owing to morning subsidence inversions. Winter concentrations were lower than summer, on average, and showed lesser diurnal variation. Temperature, wind speed, and wind direction predicted significant variability in PM_2.5_ and PM_10_ in multiple linear regression models.

**Conclusions:**

Data reveals significant morning versus afternoon variability and spatial variability in both PM_2.5_ and PM_10_ concentrations within Braddock. Information obtained on peak concentration periods, and the combined effects of industry, traffic, and elevation in this region informed the design of a larger stationary monitoring network.

## Background

Air pollution from heavy industry has decreased over recent decades, on average, in the United States [1-4]. Nonetheless, a few traditionally industrial communities remain; Braddock, PA, located east of Pittsburgh along the Monongahela River, is one such example. An economically distressed area with high rates of childhood asthma [5], Braddock is home to the Edgar Thomson Steel Works (ETSW), one of the few remaining operational steel mills owned by U.S. Steel in the Pittsburgh area. Pittsburgh became an industrial center when Andrew Carnegie sited the first steel mill along the Monongahela River in 1873. With the decline of steel industry in the early 1980s, Braddock and similar communities lost most of their economic base through layoffs, plant shutdowns, strikes, and workforce reductions [6]. The hilly terrain of the “Mon Valley” region makes measuring the spatial aspect of air pollution particularly important in Braddock.

Braddock is also situated in a federal PM_2.5_ non-attainment area [7]. The 24-h National Ambient Air Quality Standard (NAAQS) concentration (35 μg/m^3^) is typically exceeded on days of high local source emissions and inversion events, implicating both local and regional contributions. Chu et al. (2009) suggested that local pollution sources, and frequent inversion events in Pittsburgh, are superimposed on a high regional background (owing to proximity to Ohio Valley coal emissions). The extent of PM_2.5_ further varies with sunlight and photochemical processes, temperature, and wind speed and direction from more or less polluted regions [8]. The authors further hypothesized that sources southeast of Pittsburgh strongly influence PM_2.5_ on exceedance days [8], with higher concentrations likely in the source communities. The largest stationary sources of fine particles in Allegheny County lie southeast of the city -- ETSW (8.7 miles from downtown Pittsburgh) and Clairton Coke Works (14.5 miles) [9].

ETSW produced 2.7 million net tons of steel in 2010 (28% of US Steel’s domestic production) [10]. In 2008 Toxic Release Inventory (TRI) data, ETSW reported stack air releases of 33,489 lbs., primarily comprised of hydrochloric acid, ethylene, and manganese compounds, and on-site fugitive air releases were 64,849 lb (the sum of EPA Title III compounds) primarily comprised of methanol, ammonia, and zinc [11]. As a part of this mobile monitoring study, we employed Gaussian plume modeling of ETSW emissions, for neutral atmospheric conditions, which indicated centerline PM_2.5_ concentrations up to 60 μg/m^3^ within several kilometers from the plant [12]. This modeling simply provided evidence of the mill contribution to local air pollution and suggested further study in Braddock. Local PM exposures associated with ESTW, however, have not been modeled under an array of local meteorological conditions.

High outdoor air pollution, low socioeconomic status, and African American race, have all been associated with increased asthma prevalence and morbidity [13-17]. The median household income in Braddock was $26,389 in 2010 [6,18]; approximately 72% of the population is African-American. This confluence of risk factors in Braddock indicates the importance of better understanding these complex local air pollution exposure patterns, and, ultimately, the contribution of air pollution to local asthma risk [19].

There is one EPA ambient monitoring location in Braddock, at an elevation higher than that of the plant. One site, however, cannot capture fine-scale spatial variability in this complex region [20,21], or temporal patterns which may vary across space – either by elevation or by the relative predominance and location of industrial or traffic sources. As such, mobile monitoring may be an informative complement to stationary monitoring -- to better understand temporal and spatio-temporal variability [20,22] – and to help inform the spatial and temporal design of a fixed-site monitoring network for a complex region.

Mobile monitoring can be built in as a preliminary step of any air pollution field study design because it enables preliminary exploration of fine-scale spatial variability within a neighborhood, providing confidence in placement of stationary air monitors. Several characteristics of mobile monitoring facilitate its utility as a tool for understanding complex conditions, and, if carefully designed, for disentangling some aspects of temporal and spatial variation. First, mobile monitoring is cost-effective; the route can be customized to focus on particular areas of concern, such as high traffic roads or neighborhood fixed sources. Second, concentrations are typically measured at short intervals using continuous instruments which, with good quality-control efforts, can provide information about short-term peak exposures associated with adverse acute health effects [23]. Therefore, through carefully repeating time- and location-specific measures, this technique can provide some stability in determining PM concentrations. Third, mobile monitoring can also be used to validate conceptual dispersion models by capturing data at multiple points downwind of the source, under varying wind speed and direction conditions [24,25]. Finally, leveraging the repeated measures and integrating meteorology and land use characteristics, mobile monitoring data can be used to more richly characterize spatial variability throughout the region [23], by more knowledgeably tailoring the spatial and temporal characteristics of a fixed-site monitoring network.

To assess intra-community variability in pollution exposures, we collected and analyzed PM_2.5_ and PM_10_ in and around Braddock, PA, during summer 2010 and winter 2011. We repeated a mobile monitoring method along a well-characterized route, to begin to understand the within-neighborhood spatial and temporal variability. We hypothesized that PM concentrations would vary temporally according to season, time of day, day of week, and wind speed and direction. We hypothesized that PM concentrations would vary spatially by location, elevation, traffic density, and proximity to local stationary (i.e. ETSW) and mobile sources.

## Methods

### Study design

The sampling route was designed to capture variability in topography, traffic density, and proximity to ETSW. We measured PM_2.5_ and PM_10_ at specified locations along a fixed route of 25 stops using continuous instruments, during multiple weekday mornings and afternoons, in both seasons, at a range of elevations and distances from ETSW. At each location, the vehicle engine was turned off, and monitors allowed to stabilize, to obtain a stable 3- to 5-min mean concentration for each pollutant, before proceeding to the next stop. Each sampling run required approximately 3 h, during which time meteorological conditions can change significantly; for this reason, stops 1 through 5, sampled at the beginning of each sampling run, were repeated at the end (as stops 21–25).

Two sampling runs were performed through the entire route each week, during either morning or afternoon hours, to capture differing traffic patterns by time of day, and to capture potential inversion hours. Preliminary data analysis revealed, during summer afternoons, significantly less spatial or within-day variability; for this reason, subsequent runs (13–20) focused on morning hours only. In total, 20 runs (15 morning, 5 afternoon) were performed from June 3 to August 20, 2010. Twenty winter sampling runs (10 morning, 10 afternoon) were performed from November 12 to March 1, 2011.

**Figure 1:**
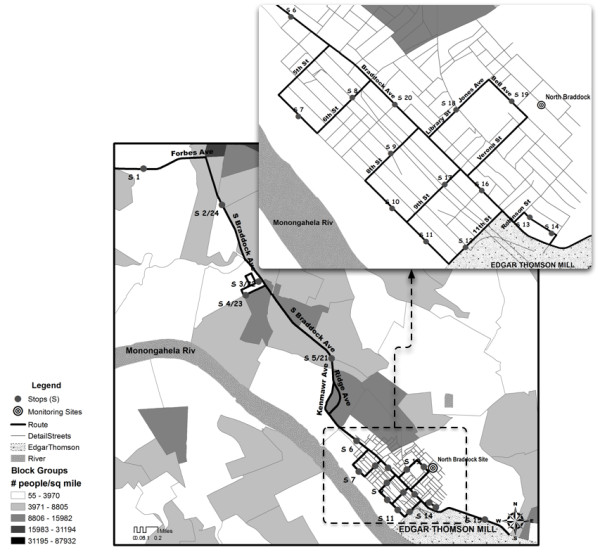
**The sampling route in Pittsburgh and Braddock, PA.** The first five stops (5 min duration) were located at higher elevation (277 to 306 m) outside of Braddock. Stops 6 through 20 (3 min duration) were located within the community of Braddock.

The sampling route began on the Carnegie Mellon University (CMU) campus location near a large city park, proceeded along a heavily trafficked urban road, and included locations near community spaces (e.g., residences, schools, churches, parks, and commercial areas), as it wound downhill into Braddock (Figure 1). Within Braddock (low-elevation sites), mobile monitoring sites were located along a road with heavy diesel truck traffic, basketball courts, an elementary school, and along the ETSW plant periphery. One designated site was in close proximity to the North Braddock EPA monitoring station, operated by Allegheny County Health Department (ACHD), shown in Figure 1.

### Monitoring instrumentation and quality control

We used the Hazdust monitor (Model EPAM-5000, Environmental Devices Corporation (EDC), Plaistow, NH 03865), a light scattering nephelometer for continuous measurements of PM_2.5_ and PM_10_. We used the EDC-supplied inlet nozzle for a cut-off of 10 μm aerodynamic diameter for PM_10_ measurements. For PM_2.5_, one Hazdust was fitted with an external size-selective inlet containing a level greased impaction surface with a cut-off of 2.5 μm (aerodynamic diameter). Both Hazdusts were calibrated to operate at 4.0 L/min, and recorded concentrations at 10-s intervals.

The Hazdust monitor automatically purges the sensor optics with clean air, and re-establishes baseline every 30 min. Prior to initiating the study, the Hazdusts were calibrated against gravimetric filter sampling by EDC using an aerosol generator with SAE fine test dust number ISO12103-1 (Arizona Road Dust), and a suite of quality assurance checks were performed. Two PM_2.5_ and two PM_10_ Hazdusts were co-located for one day; PM measures were found to correlate within 5% for each size distribution. Every five sampling runs, the four monitors were again co-located, and no significant change from the initial 5% was observed.

Before each sampling run, one PM_2.5_ and one PM_10_ monitor were secured in the backseat of a passenger vehicle. A one m PVC tube with 1.25 cm diameter was attached to each monitor, and the outlet secured 10 to 15 cm outside the rear window, on the passenger side of the car. All monitors were turned on in the parked vehicle with the engine off, and then allowed to operate for at least 20 min, or until readings stabilized, before the vehicle was turned on and the sampling route begun. At the end of this stabilization, baseline values typically ranged from 10 to 20 μg/m^3^ near the loading dock at CMU. A standardized log was used to record sampling start and stop times, unusual traffic patterns, precipitation conditions, and limited concentration information. A Colorado 400t GPS was used to mark each monitoring location, to ensure reproducibility in the precise site locations monitored during each run, and for GIS mapping and analysis.

### Covariate creation

#### Meteorological covariates

Wind speed and direction data were obtained from the ACHD Air Quality Monitoring Station in Liberty, PA at South Allegheny High School (4.9 miles South of Braddock).

Bufkit 10.11, a forecast profile visualization and analysis tool kit developed by the National Oceanic and Atmospheric Association (NOAA) and National Weather Service, was used to identify local atmospheric inversions during sampling hours, per instructions of NOAA personnel (John Darnley, personal communication). Models embedded in Bufkit include the Rapid Update Cycle (RUC), North American Mesoscale Model (NAM) and Global Forecast System (GFS). The RUC model updates once each hour, with a 1-h lag behind the latest model runs. The NAM and GFS Bufkit profiles update four times daily (0, 6, 12, and 18 h) with an approximate 2-h lag.

#### Traffic, distance to mill, and elevation covariates

An indicator of traffic density around each sampling stop was created using ESRI ArcInfo Version 10 (Redlands, CA). Roadway shapefiles for Allegheny County were obtained from Pennsylvania Department of Transportation’s (PENNDOT) publicly-available annualized average daily vehicle-count data for primary roadways. At each stop, 100-m buffers were constructed in ArcInfo, and the length of primary and secondary roads (in feet) within the buffer was calculated using open-source Geospatial Modeling Environment (Spatial Ecology LLC). Roadway lengths were multiplied by average daily traffic counts, and summed to estimate a total traffic density covariate within 100 m buffers around each of the 25 mobile monitoring stops. Secondary roadway traffic volume was estimated assuming an average daily volume of 500 vehicles, and results were sensitivity-tested for 100, 250, and 1000 vehicles/ day. Sensitivity testing for buffer size around each stop varied from 100–500 m. Sampling stops were sufficiently close together that buffers larger than 100 m overlapped, reducing apparent variability between the stops.

To evaluate the influence of topography and relative distance to ETSW, spatial covariates describing “distance to mill” and “elevation” were created using ESRI ArcInfo (version 10). Elevation above sea level was assessed as point intersection with the National Oceanographic and Atmospheric Digital Elevation Model, varying from 224 to 306 m at each of the designated stops. Using the 1996 Pennsylvania Digital Elevation Model – 10 m layer, these elevation values were calculated from the Coincident Point method using the Spatial Join tool in the Analysis Toolset [26]. Using the 2008 Allegheny County TRI Emission Points dataset, ETSW was geocoded, and distance to mill was measured as Euclidean distance between each stop and the entrance to the ETSW using the Geoprocessing Proximity Toolset ‘Near’ tool, varying between 0 to 5,633 m, with stop 12 (at entrance to the mill) designated as 0 m from ETSW [27]. ETSW was the “nearest” large source to all of the 25 designated stops.

#### Comparison to local EPA ambient monitors

Hourly PM_2.5_ and PM_10_ concentrations were obtained from ACHD. PM concentrations available from all monitors within Pittsburgh, for the specific dates and hours corresponding to the mobile monitoring runs, were analyzed and compared to mobile monitoring results.

#### Data management

Hazdust data were downloaded as .csv files, and sampling start and stop times confirmed against standardized field logs. GPS coordinates were used to confirm and map the location of each sampling event. Raw concentration data were examined for consistency across instruments and presence of outliers. Due to the inherent error of light-scattering instruments, from the raw data, outliers outside of mean +/− three standard deviations were removed, prior to deriving mean concentrations for each sampling stop. Sensitivity testing was performed to ensure consistency of results both retaining and excluding outliers. Only 1 to 2% of data (between 8–11 observations for specific PM size distribution and season) were removed, and concentration differences pre- and post-data cleaning were minimal.

#### Statistical analysis

Descriptive statistics, scatterplots, and histograms were used to characterize distributions of PM_2.5_ and PM_10_ concentrations, spatial covariates (traffic density, elevation, distance to mill) and temporal covariates (temperature, relative humidity, wind speed and direction) (Table 1). Prior to model-building, mean PM_2.5_ and PM_10_ were examined in bivariate analysis against each continuous independent variable, and between high and low categories for binary and median-dichotomized source covariates (Additional file 1). Data analysis and model-building was performed separately for PM_2.5_ and PM_10_, and for summer and winter, using only morning data (~7 am to ~10 am).

**Table 1:** Descriptive statistics for mobile monitoring morning versus afternoon

	**Summer**		**Winter**	
	***Morning***	***Afternoon***	***Morning***	***Afternoon***
Mean PM_2.5_ (μg/m^3^)	46.2	29.1	21.6	15.5
	(S.D. 35.7)	(S.D. 20.1)	(S.D. 13.5)	(S.D. 12.7)
Mean PM_10_ (μg/m^3^)	50.5	27.7	30.4	24.8
	(S.D. 37.9)	(S.D. 21.2)	(S.D. 16.5)	(S.D. 21.8)
Temperature (Mean, Min, Max °F)	70.9	78.2	23.1	35.8
	(58–80)	(68–90)	(8–50)	(17–65)
Relative Humidity (Mean, Min, Max%)	75.1	54	75.9	57.5
	(30–92)	(37–71)	(60–93)	(16–88)
Wind Speed (Mean, Min, Max mph)	5.8	7.7	5.7	9.1
	(2.9-10.4)	(6.4-10.4)	(2.9-10.4)	(2.9-16.2)

*Wind direction: Summer: 30% SW, 25% W, 15% N, 10% NW, 10% S, 5% E, 5% NE.

Winter: 35% W, 20% SW, 15% NW, 15% NE, 10% S, 5% E.

**Elevation varied from 224 to 306 m.

***Traffic density indicators varied from 595,668 to 19,961,739 based on GIS calculations around each stop (Refer to methods).

****Distance to mill varied from 0 to 5,633 m.

Multiple linear regression models were built sequentially, using a manual forward-stepwise model building procedure. Covariates significant at p < 0.05 in bivariate analysis were individually incorporated, ordered by strength of the bivariate correlation. Model fit was assessed at each stage, using the coefficient of determination (R^2^), p-value, and parameter estimate (β). At each stage, non-significant covariates were individually removed in order of descending p-value, and the model re-fit. After all significant main effects were identified and incorporated, an interaction term between wind speed and direction was examined.

Statistical analyses were conducted using Proc Reg and Proc GLM in SAS version 9.3 (SAS Institute Inc., Cary, NC) and Stata version 11 (StataCorp, LP, College Station, TX). Figures were produced using Stata 11 and SigmaPlot 10 (San Jose, CA).

#### Sensitivity testing

We examined scatterplots to assess the fit between each significant predictor and PM concentrations, to ensure that covariate selection was robust, and not reliant on outlier source values. Likewise, the fit of each additional term was tested against the residual of the prior model in the sequential model-building process. We incorporated stop order, as both an integer and categorical covariate, to identify residual within-day variance not accounted for by other temporal and meteorological covariates. We examined model residuals to ensure normality, and compared predicted PM_2.5_ and PM_10_ to observed concentrations and examined model fit through scatter plots. To assess model sensitivity to the effect of repeated measures by stop, we used the final source covariates from the linear regression model to construct a one-level mixed effects model with random effects (intercept and slope) by stop. In all cases, selected covariates retained significance, and contributed to model fit, according to Akaike information criterion (AIC).

## Results

### Summer PM_2.5_ and PM_10_ concentrations

**Figure 2:**
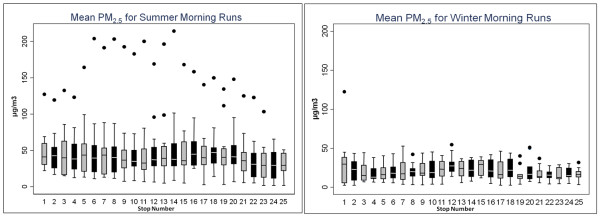
**(a) Mean PM**_**2.5**_**(μg/m**^**3**^**) for each stop from 15 summer morning runs (b) mean PM**_**2.5**_**(μg/m**^**3**^**) for each stop from 10 winter morning sampling runs.** Note that PM_2.5_ is more variable on summer mornings than on winter mornings.

**Figure 3:**
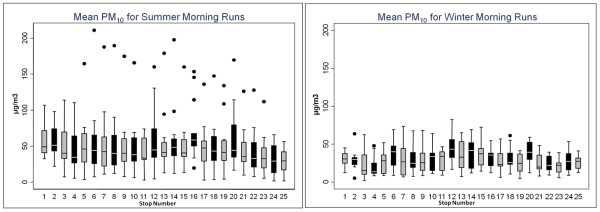
**(a) Mean PM**_**10**_**(μg/m**^**3**^**) for each stop from the 15 summer morning samplings runs and (b) mean PM**_**10**_**(μg/m**^**3**^**) for each stop on all 10 winter morning sampling runs.** A PM_10_ concentration of 319.1 μg/m^3^ was omitted for stop 11.

**Figure 4:**
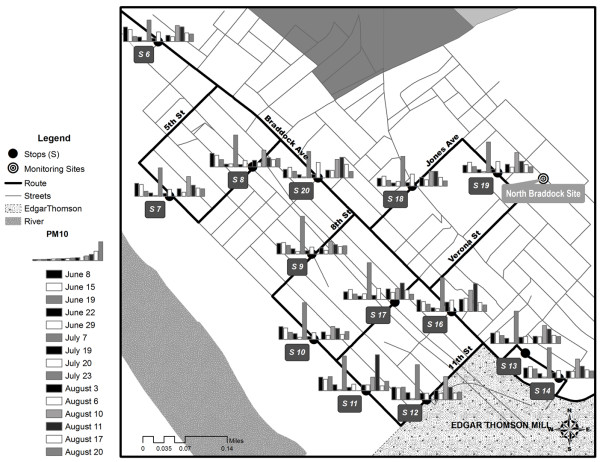
**The stops and sampled concentrations of PM**_**10**_**in downtown Braddock, PA for morning runs in the summer 2010.** The bar height refers to the average daily PM_10_ concentration (μg/m^3^) at each stop. Stop 15 (not shown) occurred further east along S. Braddock Ave.

**Figure 5:**
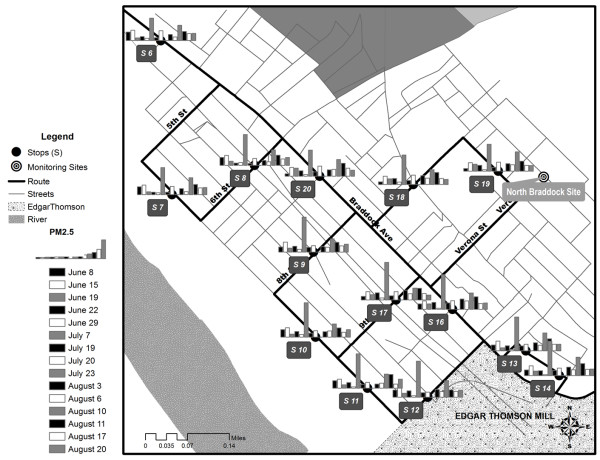
**The stops and sampled concentrations of PM**_**2.5**_**in downtown Braddock, PA for morning runs in the summer 2010.** The bar height refers to the average daily PM_2.5_ concentration (μg/m^3^) at each stop. Stop 15 (not shown) occurred further east along S. Braddock Ave.

**Figure 6:**
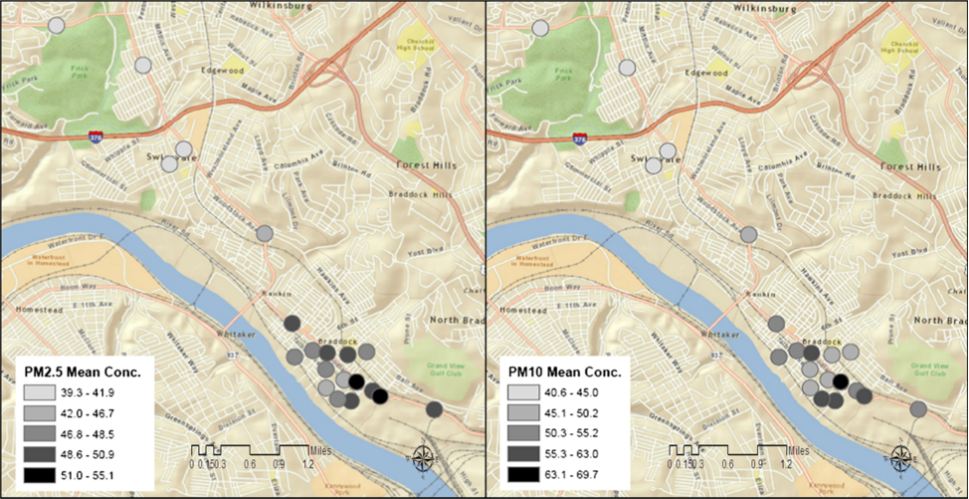
**Spatial variability of morning summer mean PM**_**2.5**_**(left) and PM**_**10**_**(right) concentrations (μg/m**^**3**^**) across all stops.**

During summer mornings, mean PM_2.5_ concentrations varied from 30.0 to 55.1 μg/m^3^ (SD = 3.3 and 13.0 μg/m^3^, respectively) across stops. Mean PM_10_ concentrations varied from 30.4 to 69.7 μg/m^3^ (SD = 2.5 and 51.2 μg/m^3^) (Figures 2 and 3). PM_2.5_ and PM_10_ mobile monitoring data depict substantial temporal variation across sampling days (Figures 4 and 5), and some spatial variation between stops (Figure 6).

**Figure 7:**
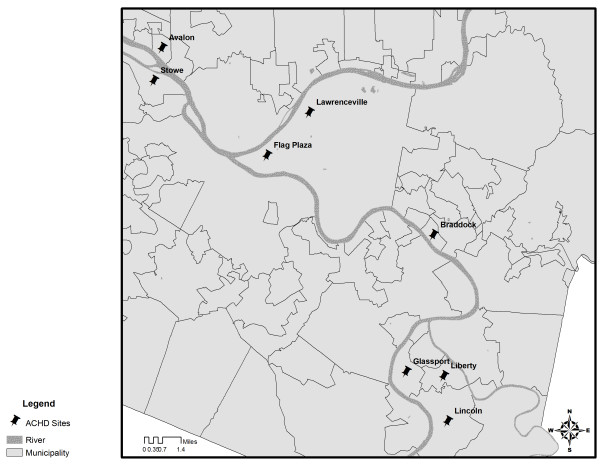
**An aerial view of the seven other Allegheny County Health Department (ACHD) monitoring sites throughout Allegheny County in relation to the city of Braddock, PA.** The North Braddock site in Figures 1, 4, 5, 6 and 7 is the Braddock ACHD site in this figure.

During summer morning sampling hours, our overall mean PM_2.5_ concentration was 46.2 μg/m^3^ (SD = 35.7 μg/m^3^), approximately twice the average concentration measured at nearby ACHD stationary monitors during the same period (Figure 7). Mean PM_2.5_ at the North Braddock monitor was 29.1 μg/m^3^ (SD = 17.9 μg/m^3^), 26.9 μg/m^3^ (SD = 12.1 μg/m^3^) at Avalon (15.0 miles from Braddock), 15.4 μg/m^3^ (SD = 8.7 μg/m^3^) at Lawrenceville (7.6 miles), and 19.9 μg/m^3^ (SD = 9.8 μg/m^3^) at Liberty (9.0 miles) during the same time period [28]. 

PM_10_ from the mobile monitors, during summer morning sampling, averaged 50.5 μg/m^3^ (SD = 37.9 μg/m^3^) overall – again approximately twice the concentrations at local ACHD monitors. PM_10_ was not collected at the North Braddock ACHD site during these hours. At Flag Plaza (downtown, 8.4 miles from Braddock) PM_10_ averaged 26.9 μg/m^3^ (SD = 11.4 μg/m^3^), at Glassport (8.4 miles) PM_10_ averaged 26.9 μg/m^3^ (SD = 15.9 μg/m^3^), at Liberty 30.0 μg/m^3^ (SD = 13.5 μg/m^3^), at Lincoln (12.0 miles) 36.6 μg/m^3^ (SD = 17.0 μg/m^3^), and at Stowe Township (14.6 miles) 28.8 μg/m^3^ (SD = 12.8 μg/m^3^).

### Morning versus afternoon concentrations, and inversion effects during summer sampling

During summer months, afternoon mobile sampling concentrations were significantly lower than morning concentrations, for both PM_2.5_ and PM_10_ (p < 0.0001) (Figures 2 and 3). The PM_2.5_ to PM_10_ ratio, during both morning and afternoon sampling, was typically above 0.8. Using BUFKIT, atmospheric inversions were identified during 50% of summer morning sampling periods. No inversion events were detected during summer afternoons or the winter sampling season. Accordingly, we observed higher PM during summer mornings than afternoons (Figures 2 and 3).

### Winter sampling data

During winter mobile air sampling, site-specific mean PM_2.5_ concentrations varied from 15.8 to 33.8 μg/m^3^ (SD = 2.4 and 11.4 μg/m^3^, respectively), and mean PM_10_ varied from 20.0 to 48.2 μg/m^3^ (SD = 2.6 and 22.5 μg/m^3^, respectively) (Figures 2 and 3). For both PM_2.5_ and PM_10_, winter concentrations were significantly lower than summer (p < 0.0001). The PM_2.5_ to PM_10_ ratio was consistently above 0.6 during winter sampling.

During winter, morning PM_2.5_ and PM_10_ concentrations were higher than afternoon, though the AM to PM difference was smaller than for summer. Mean mobile winter morning PM_2.5_ was 21.6 μg/m^3^ (SD = 13.5 μg/m^3^), but no ambient data for comparison were reported at the North Braddock monitoring site during these periods. Mean PM_10_ mobile data were 30.4 μg/m^3^ (SD = 16.5 μg/m^3^), somewhat higher than the mean ambient PM_10_ concentration of 25.9 μg/m^3^ (SD = 13.4 μg/m^3^).

### Spatial variability by proximity to local sources, elevation, and traffic

**Figure 8:**
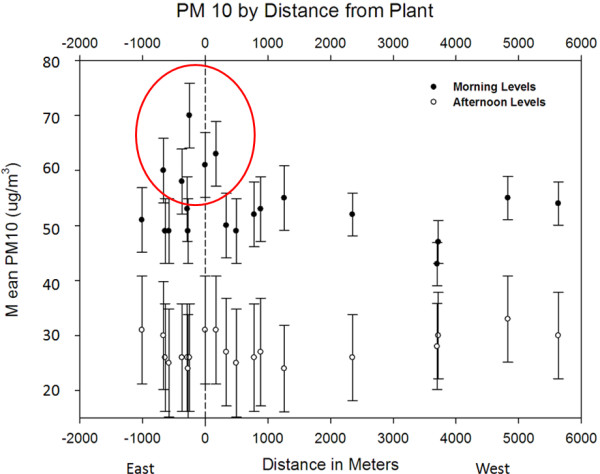
**Measured summer PM**_**10**_**concentrations (μg/m**^**3**^**) based on distance of stop from the plant.** The circled points are likely fugitive emissions or road dust from truck traffic. This trend was not observed for PM_2.5_ (Additional file 1). Stop 12 was used as the distance 0 since it was at the gate of ETSW. Note the clear difference between morning and afternoon runs.

During morning sampling, PM_10_ was relatively higher at a cluster of stops near the plant (Stops 11, 12, 13, 14, and 16), indicating near-source spatial variability for PM_10_ (Figure 8). A similar plot for PM_2.5_ indicated no elevated concentrations near the plant (Additional file 1).

In Braddock, mean PM_2.5_ concentrations at higher elevation (stops 18 and 19) did not significantly differ from concentrations at lower elevations (i.e. along South Braddock Avenue in downtown Braddock). Mean PM_10_, however, was significantly lower at stops 18 and 19, compared to lower-elevation stops closer to ETSW.

PM_2.5_ and PM_10_ concentrations did not significantly correlate with traffic density, either during summer or winter, morning or afternoon sampling (p > 0.20 in all cases).

### Temporal variation within the sampling period

To assess the effect of temporal variation during each sampling period, five stops were sampled at both the beginning and end of each run, labeled as stops 1–5 and 25–21, respectively. Pairwise comparison of these repeated stops suggests a significant temporal effect during summer morning sampling, with lower concentrations of both PM_2.5_ and PM_10_ later in the morning (p < 0.029); repeated stops did not significantly differ in the winter (p > 0.169).

### Wind data and photochemical smog

In bivariate analysis, wind speed was positively correlated with PM_2.5_ concentrations during both summer and winter mornings, and PM_10_ concentrations during the summer (p < 0.05) (Additional file 1). Wind direction also significantly affected concentrations; higher PM_2.5_ were observed during periods when winds blew from the south and southwest, and lower concentrations when winds blew from the north or northeast, relative to periods of easterly winds. For PM10, concentrations were significantly higher during periods of non-easterly winds, with lower concentrations only with winds from the north (Table 2).

**Table 2:** **Final mixed model covariates and model fits for summer morning PM**_**2.5**_**and PM**_**10**_

		**Mixed model**		
Summer Morning PM_2.5_ (μg/m^3^)	**Covariates**	β (SE)	p-value	Seq R^2^*
	Intercept	102.63 (16.364)	<.0001	--
	Temperature (°F)	−1.99 (0.297)	<.0001	0.15
	Wind speed (mph)	21.83 (2.120)	<.0001	0.27
	Wind Direction (blowing from):		<.0001	0.58
	*W*	23.885 (3.435)	<.0001	--
	*S*	158.33 (12.284)	<.0001	--
	*SW*	109.09 (5.199)	<.0001	--
	*SE*	8.406 (3.154)	0.0080	--
	*N*	−48.763 (12.322)	<.0001	--
	*NE*	−115.40 (11.904)	<.0001	--
	*E*	0	--	--
	Distance to mill (m)	−0.00145 (.00034)	<.0001	0.59
	Wind speed x Wind direction**:	--	<.0001	**0.74**
	*N*	−39.674 (3.112)	<.0001	--
	*S*	6.264 (2.625)	0.0176	--
Summer Morning PM_10_ (μg/m^3^)	Intercept	−37.949 (8.932)	0.0066	--
	Wind speed (mph)	23.896 (2.683)	<.0001	0.17
	Wind Direction (blowing from):		<.0001	0.46
	*W*	32.833 (5.476)	<.0001	--
	*S*	110.52 (10.786)	<.0001	--
	*SW*	80.831 (5.590)	<.0001	--
	*SE*	2.866 (4.377)	0.5130	--
	*N*	−29.902 (8.534)	<.0001	--
	*NE*	−135.66 (16.140)	<.0001	--
	*E*	0	--	--
	Distance to mill (m)	−0.0022 (.0005)	<.0001	0.48
	Inversion presence	13.634 (2.960)	<.0001	0.53
	Wind speed x Wind direction**:	--	<.0001	**0.64**
	*N*	−30.397 (2.960)	<.0001	--

*Seq R^2^ is the sequential model fit for each additional term incorporated into model.

**Only significant effects shown in wind speed x wind direction interaction.

***t-tests for coefficients of the model and the F-test p-values were all <0.001, and the Durbin-Watson test found no collinearity.

On July 7, an inversion event that contributed to a photochemical smog occurred in and around the Pittsburgh area, during a morning sampling run. Mean PM_2.5_ measured between 46.2 and 214.3 (SD = 10.7) μg/m^3^ across all stops, and mean PM_10_ measured between 42.4 and 319.1 (SD = 31.1) μg/m^3^. During this period, elevated PM_10_ concentrations were noted at the local ambient monitors: Glassport (58.4 μg/m^3^ (SD = 32.7 μg/m^3^)), Lincoln (61.8 μg/m^3^ (SD = 32.4 μg/m^3^)), Liberty (55 μg/m^3^ (SD = 28.2 μg/m^3^)), and Stowe Township (52.8 μg/m^3^ (SD = 42.2 μg/m^3^)).

### Multiple linear regression model building

Pearson correlations between covariates and PM concentrations were determined (Additional file 1). During summer mornings, PM_2.5_ was predominantly explained by meteorology – temperature, wind speed, wind direction, and the interaction of wind speed and direction (Temporal Seq R^2^ = 0.73, Spatial R^2^ = 0.002) (Table 2). Higher temperatures conferred lower concentrations, after adjusting for all other model terms; each additional degree F was associated with a decrease of about 2 μg/m^3^ in PM_2.5_ (a major determinant of mixing height is air temperature). Higher wind speeds, on average, were associated with higher PM_2.5_, though this effect was strongly modified by wind direction; higher concentrations were observed with winds from the south or southwest (from direction of mill or Ohio Valley, respectively), and lower concentrations from the north or northeast. Distance to mill was the only spatial covariate that explained additional (marginal) variability in PM_2.5_ during summer mornings; PM_2.5_ was 0.145 μg/m^3^ lower, on average, for each 100 m distance from the mill. Overall model fit was strong, explaining approximately 74% of the variability in PM_2.5_ concentrations.

For summer mornings, PM_10_ was also predominantly explained by meteorology, including terms for wind speed, wind direction, inversion events, and an interaction between wind speed and direction (Temporal Seq R^2^ = 0.62, Spatial R^2^ = 0.01) (Table 2). Notably, both wind speed and direction had similar influences on PM_10_ as on PM_2.5_. PM_10_ was 0.220 μg/m^3^ lower, on average, with each 100 m distance from the mill. The presence of inversions accounted for an increase of 13.6 μg/m^3^, on average, in PM_10_. Overall model fit was strong, explaining approximately 64% of the variability in PM_10_ concentrations. The models for winter sampling were similar to summer models (explaining 51 to 54% of variability in PM), as meteorology was the primary contributor and elevation replaced distance from the mill (data not shown).

In sensitivity testing, final model results were robust to the effects of outliers and repeated measures by stop. All model covariates retained significance regardless of other terms retained in each model, and contributed to model fit, according to Akaike information criterion (AIC). Stop order was incorporated as a sensitivity analysis, to identify additional within-day variance not captured by temporal or meteorological terms, but did not improve model fit.

## Discussion

Our measurements of PM_2.5_ and PM_10_ concentrations in and around Braddock, PA, during summer and winter months 2010–2011, highlight the impact of summer morning inversion events on particulate pollution. PM concentrations showed a temporal pattern, but were relatively spatially homogenous for our sampling routes. We observed large temporal variation in short term measured PM_2.5_ and PM_10_ across multiple sampling days, including higher PM_2.5_ and PM_10_ concentrations in summer vs. winter and morning vs. afternoon. These findings provide a better understanding of the spatial and temporal variability of PM in Braddock, and provided critical information about appropriate sampling windows for future monitoring.

During summer, patterns were observed between morning and afternoon PM concentrations. The PM ratio was above 0.8 for summer sampling, suggesting fresh fine plant-related particle emissions (e.g. furnace and trucks), in contrast to re-suspension at the sampling sites; the PM ratio was above 0.6 for winter sampling, and salt spread on the street may have contributed to re-suspended PM [29,30]. Data on this PM ratio are sparse, and the National Resources Defense Council (NRDC) assumes this ratio is typically 60% in US cities. [31]. Though most influence appeared to be from fine particles, the main influence of PM_10_ occurred in areas directly adjacent to the plant facilities during the morning. Distance to the mill was a significant covariate in the summer sampling session (Table 2). Further, the decline of PM_10_ as one moved away from the plant into the community was an important spatial result for the future stationary monitoring campaign throughout Braddock (Figure 8). The spatially-created traffic variables were insignificant in the regression modeling including all stops. However, when comparing a stop that was repeated the same day over a time differential of approximately 3 h, significant differences were seen in PM concentration and traffic may be a contributor to those changes.

The current study demonstrated that spatial and temporal relationships need to be determined in a first step to adequately characterize exposure of individuals living and working in the Braddock area. These findings provide a better understanding of air pollution exposure patterns around Braddock, PA, which may have important public health and policy repercussions [32,33].

Important factors included topography (i.e. elevation) and local atmospheric inversions. Elevation was a significant covariate for the winter sampling session. Fine PM in this urban area was also influenced by proximity to the steel mill transient emission events, [34]. During a temperature inversion, the air becomes stagnant, and the valley walls trap air pollution near the surface. Inversion was included for the summer morning PM_10_ model, but dropped out of significance for the PM_2.5_ model when the wind interaction term was incorporated (Table 2). For summer sampling, stops 21 to 25 typically recorded PM_2.5_ and PM_10_ concentrations lower than those measured at stops 1 to 5. It is likely that the observed variations are due to changes in the influences of sources. Chu et al. (2009) reported that sources to the south and southeast of the Pittsburgh Supersite significantly influenced PM_2.5._ Sources located in other directions from the monitoring site had less influence despite greater emissions and a high frequency of winds. Building on Chu et al. (2009), we examined the role of wind. In assessing our multiple linear regression models, wind direction appeared to be the strongest covariate for the summer and winter months. Winds have been shown to play important roles in transport of pollutants, such as photochemical transport from New York City into Connecticut [35]. Wind speed was positively correlated with PM_2.5_ concentrations during both summer and winter mornings, even though wind speed is generally negatively correlated with air pollutants. However, since the meteorology is measured at an away location in Liberty, PA, local perturbations due to dilution of primary particles from the sources could have been masked by area sources. Chu et al. (2010) demonstrated that high temperatures and relative humidity in the eastern United States may be associated with high PM_2.5_ concentrations to a greater extent than elevated concentrations of SO_2_ or O_3_ or high levels of UV. We did not find association with relative humidity (RH), but an inverse relationship with temperature (higher temperatures resulted in lower PM) was found in models (Table 2). One possibility for higher PM in the summer could be power plant emissions, but more likely in the eastern US it is a higher baseline caused by secondary aerosols formed by photochemical smog. A higher temperature would have broken up an inversion, resulting in lower PM concentrations from local sources; mobile monitoring occurred at specific times of the day (morning versus afternoon hours), so hourly temperature data were used instead of 24 hour average temperatures.

A strength of the mobile monitoring approach is that it allowed us to construct multiple snapshots of spatial and temporal variability in air pollution in areas immediately adjacent to mobile or stationary sources relatively quickly and inexpensively. It also provided a detailed morning versus afternoon pattern in PM concentrations for the summer months 2010, and suggested that fresh combustion and particle re-suspension may be the primary sources for PM pollution in and around Braddock. In contrast to prior mobile monitoring studies, we instituted a practice to account for session temporal variability by re-sampling the same stops at the beginning and end of the route. A criticism of many studies that aim to discover a relationship between air pollution and health is that exposure is typically characterized using measurements from a few sparsely located air quality monitoring stations, and often only one [36]. Mobile monitoring has been used to characterize spatial variability in black carbon concentrations for land use regression, even though spatial modeling conventionally requires longer-term measurements at multiple locations [23]. Conversely, our mobile monitoring approach provided preliminary insight towards understanding spatial and temporal exposure variation throughout the Braddock area.

Because the mobile monitoring devices are handheld, cost-effective (e.g. multiple samples with high frequency and mobility), and can provide real-time PM or VOC measurements, there is a possibility that communities could deploy these units after a training program conducted by skilled exposure or air pollution scientists [37,38]. Active neighborhood sampling could improve residents’ knowledge about local air pollution concentrations, and enable residents to investigate areas where air pollution is perceived to be elevated. By following a time- and location-specific approach, communities could collect a significant amount of repeated measures data to better understand pollution concentrations where they reside, and to identify high-pollution events. Therefore, mobile monitoring could be investigated for use in community based participatory research (CBPR) to provide neighborhood residents with the opportunity to proactively investigate potential air pollution. However, interpretation of the results will still require skilled professional analysis.

While the mobile monitoring data provided valuable information, one limitation is that a sampling interval of 3 to 5 min is too short to provide an accurate exposure profile for Braddock residents; these data primarily allow us to gain an understanding of patterns of exposure, and future studies can then be designed to better elucidate stable patterns in exposure variation, and examine associations with asthma and other chronic disease outcomes. Although ETSW operates year round, specific plant activity data would have been important in explaining the temporal variation between sampling days, but data was not available for analyses. Future monitoring will include sites with a more complete contrast in source proximity, elevation, and density of traffic, with a specific interest in morning sampling (6 AM to 11 AM), a design that results from this study, to observe potential effects of inversion events on air pollution concentrations across the Pittsburgh region. A technological limitation is that the Hazdust EPAM-5000 is calibrated using “Arizona road dust” (EDC, Plaistow, New Hampshire -personal communication), which is not representative of Pittsburgh-area aerosols. For this reason, comparisons were provided between our data and ACHD federal reference method (FRM) measurements in Pittsburgh. However, it is difficult to calibrate any continuous monitor with the local aerosol because this would require resuspending the material, changing its basic character and size distribution.

Our approach provided the foundation for the design of a longer-term air pollution monitoring strategy for Braddock and the city of Pittsburgh. Based on results from this study, city-wide sampling will be performed Monday through Friday during potential morning inversion hours (6 to 11 AM) using eight stationary monitors (two reference monitors, six distributed monitors), randomized and spatially re-allocated each week, over six weeks each season, to estimate PM_2.5_ in concentrations capturing the range of elevation, proximity to industry, and traffic density across the Pittsburgh metropolitan area.

## Conclusions

In an effort to characterize PM concentrations in and around Braddock, we identified a seasonal and morning versus afternoon pattern in PM concentrations and observed variability of PM over space and time with a strategically-designed mobile sampling protocol. Summertime continuous monitoring led to higher levels compared to the winter, and PM_10_ levels were elevated in the area near the Edgar Thomson Steel Works. The results point to plant operations-related particle emissions as the primary source for PM pollution in and immediately around Braddock. Future research will build upon these data and include a dense stationary monitoring campaign in Pittsburgh in which spatial and temporal variability of PM will be assessed to further understand air pollution exposures.

## Abbreviations

ETSW: Edgar Thomson Steel Works; PM2.5: Particulate Matter less than 2.5 μm in diameter; PM10: Particulate Matter less than 10 μm in diameter; NAAQS: National Ambient Air Quality Standard; TRI: Toxic Release Inventory; EPA: Environmental Protection Agency; ACHD: Allegheny County Health Department; EDC: Environmental Devices Corporation; GIS: Geographic Information System; NOAA: National Oceanic and Atmospheric Association.

## Competing interests

The authors declare that they have no competing interests.

## Authors' contributions

BT carried out the mobile monitoring studies, participated in statistical analyses, and drafted the manuscript. PL has been involved in drafting the manuscript and revising it critically for important intellectual content. JC has been involved in drafting the manuscript and revising it critically for important intellectual content. KS has been involved in drafting the manuscript and revising it critically for important intellectual content, carried out the mobile monitoring studies, participated in statistical analyses. CD has been involved in drafting the manuscript and revising it critically for important intellectual content. JK has been involved in drafting the manuscript and revising it critically for important intellectual content. NC carried out the mobile monitoring studies. JZ carried out the mobile monitoring studies. GP participated in statistical analyses. BP participated in statistical analyses. FH has been involved in drafting the manuscript and revising it critically for important intellectual content. All authors read and approved the final manuscript.

## Supplementary Material

Additional file 1 **Descriptive statistics for PM**_**2.5 **_**and PM**_**10 **_**(summer morning runs only), June to August 2010.** Click here for file
