# Supplementary material for: Understanding intra-neighborhood patterns in PM2.5 and PM10 using mobile monitoring in Braddock, PA
Source: Environ Health. 2012 Oct 10;11:76. doi: 10.1186/1476-069X-11-76 (PMC3639095; doi:10.1186/1476-069X-11-76)
Supplement: Additional file 1 — Descriptive statistics for PM2.5 and PM10 (summer morning runs only), June to August 2010. [file 1476-069X-11-76-S1.docx]

**Supplemental Material**

Table 1S. Descriptive statistics for PM_2.5_ and PM_10_ (summer morning runs only),

June to August 2010.

*p-values used to compare repeated stops (Stop 1 vs Stop 25, Stop 2 vs

Stop 24, Stop 3 vs Stop 22, Stop 4 vs Stop 23, Stop 5 vs Stop 21).

|  | **PM_2.5_ (µg/m^3^)** | | | | **PM_10_ (µg/m^3^)** | | | |
| --- | --- | --- | --- | --- | --- | --- | --- | --- |
| **Stop** | **n** | **Mean** | **SD** | **p-value** | **n** | **Mean** | **SD** | **p-value** |
| 1 | 15 | 48.9 | 4.0 |  | 15 | 56.3 | 5.1 |  |
| 2 | 15 | 47.2 | 3.4 |  | 15 | 55.5 | 3.7 |  |
| 3 | 15 | 46.5 | 4.2 |  | 14 | 47.2 | 2.9 |  |
| 4 | 15 | 45.3 | 3.4 |  | 14 | 43.2 | 2.9 |  |
| 5 | 15 | 52.0 | 3.9 |  | 14 | 51.9 | 3.4 |  |
| 6 | 15 | 49.7 | 5.0 |  | 14 | 55.2 | 7.8 |  |
| 7 | 15 | 47.7 | 4.6 |  | 14 | 53.3 | 4.6 |  |
| 8 | 15 | 48.5 | 3.7 |  | 14 | 52.4 | 3.7 |  |
| 9 | 15 | 47.7 | 3.3 |  | 14 | 49.1 | 3.4 |  |
| 10 | 15 | 46.5 | 4.1 |  | 14 | 50.2 | 6.8 |  |
| 11 | 15 | 48.1 | 13.0 |  | 14 | 63.0 | 51.2 |  |
| 12 | 15 | 49.1 | 8.1 |  | 14 | 60.7 | 13.3 |  |
| 13 | 15 | 50.9 | 5.1 |  | 14 | 53.4 | 4.0 |  |
| 14 | 15 | 55.1 | 4.6 |  | 14 | 57.6 | 5.5 |  |
| 15 | 15 | 49.4 | 3.9 |  | 14 | 51.4 | 4.9 |  |
| 16 | 15 | 54.3 | 9.8 |  | 14 | 69.7 | 26.8 |  |
| 17 | 15 | 45.8 | 3.7 |  | 14 | 48.8 | 4.6 |  |
| 18 | 15 | 50.2 | 12.6 |  | 14 | 48.5 | 2.9 |  |
| 19 | 15 | 47.4 | 4.8 |  | 14 | 49.4 | 4.3 |  |
| 20 | 15 | 49.6 | 7.1 |  | 14 | 59.7 | 11.9 |  |
| 21 | 15 | 41.4 | 3.4 | **0.027** | 14 | 45.1 | 2.6 | 0.143 |
| 22 | 15 | 37.2 | 3.5 | **0.029** | 14 | 42.8 | 3.1 | 0.343 |
| 23 | 15 | 34.3 | 4.2 | **0.011** | 14 | 37.9 | 2.5 | 0.125 |
| 24 | 15 | 31.3 | 4.0 | **0.005** | 14 | 30.5 | 2.8 | **0.001** |
| 25 | 15 | 30.0 | 4.1 | **0.005** | 14 | 30.4 | 3.7 | **0.001** |
| **Overall** | 375 | 46.2 | 5.2 |  | 352 | 50.6 | 7.5 |  |

Table 2S. Descriptive statistics for PM_2.5_ and PM_10_ (winter morning runs only),

November 2010 to March 2011.

*p-values used to compare repeated stops (Stop 1 vs Stop 25, Stop 2 vs

Stop 24, Stop 3 vs Stop 22, Stop 4 vs Stop 23, Stop 5 vs Stop 21).

|  | **PM_2.5_ (µg/m^3^)** | | | | **PM_10_ (µg/m^3^)** | | | |
| --- | --- | --- | --- | --- | --- | --- | --- | --- |
| **Stop** | **n** | **Mean** | **SD** | **p-value** | **n** | **Mean** | **SD** | **p-value** |
| 1 | 10 | 33.8 | 9.1 |  | 10 | 31.1 | 3.7 |  |
| 2 | 10 | 23.2 | 4.2 |  | 10 | 29.3 | 5.3 |  |
| 3 | 10 | 20.2 | 3.6 |  | 10 | 22.2 | 4.5 |  |
| 4 | 10 | 18.0 | 3.4 |  | 10 | 20.8 | 4.6 |  |
| 5 | 10 | 19.5 | 4.4 |  | 10 | 25.7 | 3.2 |  |
| 6 | 10 | 21.4 | 4.1 |  | 10 | 36.4 | 7.9 |  |
| 7 | 10 | 22.1 | 11.4 |  | 10 | 30.3 | 3.4 |  |
| 8 | 10 | 21.0 | 2.7 |  | 10 | 29.3 | 4.6 |  |
| 9 | 10 | 21.8 | 3.1 |  | 10 | 28.9 | 2.9 |  |
| 10 | 10 | 22.9 | 2.4 |  | 10 | 31.5 | 4.3 |  |
| 11 | 10 | 23.3 | 4.2 |  | 10 | 30.4 | 2.9 |  |
| 12 | 10 | 29.5 | 11.2 |  | 10 | 48.2 | 22.5 |  |
| 13 | 10 | 23.4 | 3.5 |  | 10 | 35.3 | 3.3 |  |
| 14 | 10 | 23.5 | 2.9 |  | 10 | 37.4 | 2.6 |  |
| 15 | 10 | 26.0 | 5.4 |  | 10 | 38.8 | 7.8 |  |
| 16 | 10 | 23.0 | 5.5 |  | 10 | 33.0 | 8.9 |  |
| 17 | 10 | 21.0 | 3.2 |  | 10 | 28.1 | 5.0 |  |
| 18 | 10 | 23.2 | 7.3 |  | 10 | 32.7 | 7.5 |  |
| 19 | 10 | 16.7 | 2.7 |  | 10 | 24.8 | 3.0 |  |
| 20 | 10 | 22.3 | 5.2 |  | 10 | 40.4 | 10.0 |  |
| 21 | 10 | 17.6 | 2.7 | 0.374 | 10 | 25.7 | 5.1 | 0.773 |
| 22 | 10 | 16.2 | 3.7 | 0.383 | 10 | 24.6 | 3.6 | 0.738 |
| 23 | 10 | 15.8 | 2.5 | 0.566 | 10 | 20.0 | 3.2 | 0.703 |
| 24 | 10 | 17.6 | 2.8 | 0.248 | 10 | 27.9 | 9.3 | 0.655 |
| 25 | 10 | 17.1 | 3.3 | 0.169 | 10 | 25.4 | 4.9 | 0.432 |
| **Overall** | 250 | 21.6 | 4.6 |  | 250 | 30.4 | 5.7 |  |

Table 3S. Pearson correlations between covariates and PM concentrations

| **Covariates** | **Summer Morning PM_2.5_ (µg/m^3^)** | **Summer Morning PM_10_**  **(µg/m^3^)** | **Winter Morning PM_2.5_**  **(µg/m^3^)** | **Winter Morning PM_10_**  **(µg/m^3^)** |
| --- | --- | --- | --- | --- |
| *Spatial:* |  |  |  |  |
| Traffic density | <0.001 | <0.001 | 0.002 | 0.01 |
| Elevation (m) | <0.001 | 0.01 | **0.03** | **0.05** |
| Distance to mill (m) | 0.0017 | 0.01 | **0.02** | **0.04** |
| *Temporal:* |  |  |  |  |
| Temperature (°F) | **0.15** | **0.06** | **0.25** | **0.16** |
| Relative Humidity | **0.04** | **0.12** | 0.06 | 0.02 |
| Wind speed (mph) | **0.04** | **0.09** | **0.03** | <0.001 |
| Wind direction* | **0.49** | **0.44** | **0.38** | **0.24** |
| Inversion presence | **0.03** | **0.06** | **N/A | **N/A |

*Categorical variables were created for wind direction.

**No inversion events detected in winter sampling.

Table 4S. Final Mixed Model covariates and model fits for winter morning PM_2.5_ and PM_10._

|  |  | **Mixed Model** | | |
| --- | --- | --- | --- | --- |
|  | **Covariate description** | β (SE) | p-value | Seq R^2^ |
| Winter Morning PM_2.5_  (µg/m^3^) | Intercept | 5.899 (8.038) | 0.4637 | -- |
|  | Temperature (°F) | -1.959 (0.340) | <.0001 | 0.25 |
|  | Wind speed (mph) | 6.750 (1.169) | <.0001 | 0.25 |
|  | Site Wind direction: |  | <.0001 | 0.42 |
|  | *E E* | 87.865 (12.151) | <.0001 | -- |
|  | *NE* | 42.795 (7.964) | <.0001 | -- |
|  | *NW* | 49.339 (9.593) | <.0001 | -- |
|  | *S* | -121.40 (18.543) | <.0001 | -- |
|  | *SW* | 52.125 (9.358) | <.0001 | -- |
|  | *W* | 0 | -- | -- |
|  | Elevation (m) | -0.0695 (0.0194) | 0.0004 | 0.44 |
|  | Wind speed x Wind direction: | -- | <.0001 | **0.54** |
|  | *S* | 24.015 (3.425) | <.0001 | -- |
| Winter Morning PM_10_ (µg/m^3^) | Intercept | -0.551 (12.470) | 0.9648 | -- |
|  | Temperature (°F) | -4.154 (0.473) | <.0001 | 0.16 |
|  | Wind speed (mph) | 13.498 (1.717) | <.0001 | 0.16 |
|  | Wind direction: |  | <.0001 | 0.26 |
|  | *E* | 159.50 (17.417) | <.0001 | -- |
|  | *NE* | 97.496 (11.835) | <.0001 | -- |
|  | *NW* | 110.48 (13.984) | <.0001 | -- |
|  | *S* | -226.06 (26.480) | <.0001 | -- |
|  | *SW* | 120.93 (13.672) | <.0001 | -- |
|  | *W* | 0 | -- | -- |
|  | Elevation (m) | -0.135 | <.0001 | 0.31 |
|  | Wind speed x Wind direction: | -- | <.0001 | **0.51** |
|  | *S* | 46.058 (4.805) | <.0001 | -- |

*t-tests for coefficients of the model and the F-test p-values were all <0.001, and the Durbin-Watson test found no collinearity.


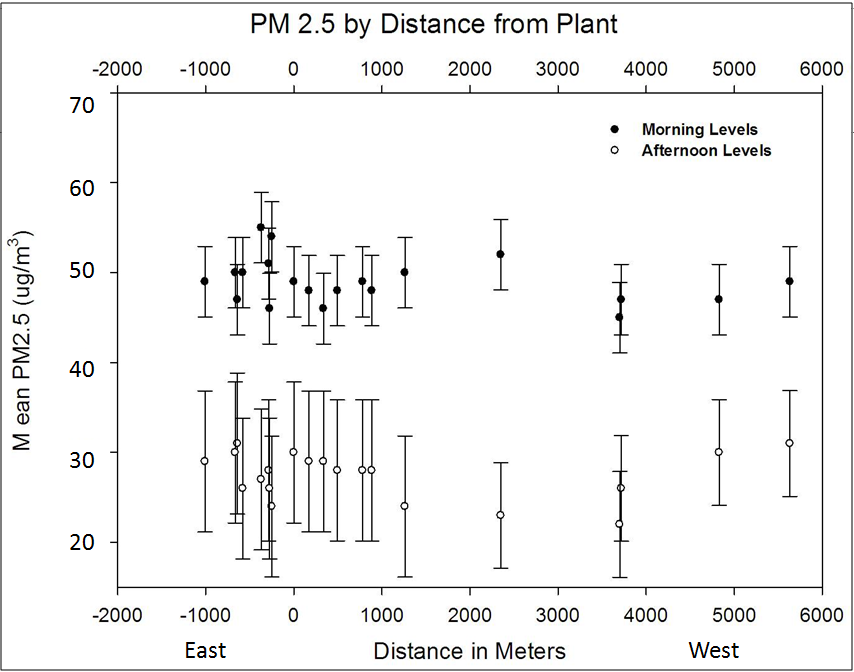


Figure 1S. Measured summer PM_2.5_ concentrations (µg/m^3^) based on distance of stop for the plant. Stop 12 was used as distance 0 since it was at the gate of ETSW. Note the clear difference between morning and afternoon runs.
